# Effects of a smartphone-based videoconferencing program for older nursing home residents on depression, loneliness, and quality of life: a quasi-experimental study

**DOI:** 10.1186/s12877-020-1426-2

**Published:** 2020-01-28

**Authors:** Hsiu-Hsin Tsai, Ching-Yu Cheng, Wann-Yun Shieh, Yue-Cune Chang

**Affiliations:** 1 School of Nursing, College of Medicine, Chang Gung University, 259, Wen-Hwa 1st Road, Kwei-Shan, Tao-Yuan, Republic of China; 2 Department of Psychiatry, Chang Gung Memorial Hospital at Linkou, Tao-Yuan, Republic of China; 3 College of Nursing, Chang Gung University of Science and Technology, Chia-Yi, Republic of China; 4 Research Fellow, Chang Gung Memorial Hospital at Chiayi, Chai-Yi, Republic of China; 5 Department of Computer Science and Information Engineering, Chang Gung University, Tao-Yuan, Republic of China; 6 Department of Mathematics, Tamkang University, New Taipei City, Republic of China

**Keywords:** Videoconferencing, Smartphone, Quality of life, Depression, Loneliness

## Abstract

**Background:**

Smartphones can optimize the opportunities for interactions between nursing home residents and their families. However, the effectiveness of smartphone-based videoconferencing programs in enhancing emotional status and quality of life has not been explored. The purpose of this study was to evaluate of the effect of a smartphone-based videoconferencing program on nursing home residents’ feelings of loneliness, depressive symptoms and quality of life.

**Methods:**

This study used a quasi-experimental research design. Older residents from seven nursing homes in Taiwan participated in this study. Nursing homes (NH) were randomly selected as sites for either the intervention group (5 NH) or the control group (2 NH); NH residents who met the inclusion criteria were invited to participate. The intervention group was comprised of 32 participants; the control group was comprised of 30 participants. The intervention group interacted with their family members once a week for 6 months using a smartphone and a “LINE” application (app). Data were collected with self-report instruments: subjective feelings of loneliness, using the University of California Los Angeles Loneliness Scale; depressive symptoms, using the Geriatric Depression Scale; and quality of life using the SF-36. Data were collected at four time points (baseline, and at 1-month, 3-months and 6-months from baseline). Data were analysed using the generalized estimating equation approach.

**Results:**

After the intervention, as compared to those in the control group, participants in interventional group had significant decreases in baseline loneliness scores at 1 months (β = − 3.41, *p* < 0.001), 3 months (β = − 5.96, *p* < 0.001), and 6 months (β = − 7.50, *p* < 0.001), and improvements in physical role (β = 36.49, *p* = 0.01), vitality (β = 13.11, *p* < 0.001) and pain scores (β = 16.71, *p* = 0.01) at 6 months. However, changes in mean depression scores did not significantly differ between groups.

**Conclusions:**

Smartphone-based videoconferencing effectively improved residents’ feelings of loneliness, and physiological health, vitality and pain, but not depressive symptoms. Future investigations might evaluate the effectiveness of other media-based technologies in nursing homes as well as their effectiveness within and between different age cohorts.

## Background

Placement of older adults in a nursing home (NH) can be a stressful life event that produces a higher prevalence of depression [1–3], loneliness, and a reduction in quality of life (QoL) [4, 5]. Depression is a leading cause of disability worldwide and feelings of loneliness contribute significantly to depression, resulting in reductions in QoL [6–8]. NH residents often feel isolated, which can further increase feelings of loneliness and depression. Therefore, developing interventions for NH residents that can reduce feelings of loneliness and depression could improve their quality of life.

Providing social support for NH residents is important because the quality and quantity of social support systems used by older people are closely related to both their health status and QoL [5, 9]. One important aspect of such emotional support for older NH residents is the continuous involvement of their family members. This involvement is something especially vital in the Chinese culture where families play a salient role in maintaining residents’ emotional well-being.

However, one-third of NH residents rarely have visitors [10]; and only about 45–60% of the NH residents’ families visit them once a week [11, 12]. A longitudinal study revealed that there is a tendency for family visits to decline slightly over time [13]. Providing weekly desktop-based videoconferencing to NH residents was shown to effectively reduce depression and general feelings of loneliness among Taiwanese NH residents [12, 14]. Nevertheless, no research has explored the effect of videoconferencing on residents’ QoL. Furthermore, the size and weight of desktop-based equipment may limit their convenience. Yet, with the development of technology, smartphones have become a particularly attractive modality for delivering health related interventions because of their widespread adoption and increasingly powerful technical capabilities. Unlike desktop computers or even laptops, smartphones are hand-held devices [15]. Such a technology can, accordingly, support daily life activities, address safety issues, and provide communication and entertainment. Compared to desktop devices, smartphones may optimize the opportunities for interactions between NH residents and their families. However, the effectiveness of this medium in enhancing QoL and emotional status for older adults has not been evaluated. Consequently, the lack of empirical evidence on videoconferencing for NH residents provides ample justification for examining the impact of this modality more fully. Therefore, the purpose of this study, was to evaluate the effectiveness of a smartphone-based videoconference program on NH residents’ loneliness, depressive symptoms and QoL.

## Methods

### Design, sample, and setting

A quasi-experimental design was employed. Participants were recruited from seven nursing homes in Taiwan. The NH were purposively sampled based on size (medium-large, > 65 beds) and located within easy reach of the researchers [16]. Each NH was randomly assigned to provide participants for either the intervention or control group. Assuming a power of .80, alpha value of .05, and moderate effect size of t-test (.05), it was estimated that 26 participants were needed for two groups (experimental and control). We then factored in an estimated 15 to 20% attrition rate, due to mortality. Each NH was assigned a number and potential participants from each NH were randomly selected until we achieved a target of 30 participants in each group [17].

Residents were recruited if they: 1) were over 60 years of age; 2) had a Mini-Mental State Examination (MMSE) score ≥ 16 for residents with no formal education or MMSE> 20 for residents with at least a primary school education [18, 19]; 3) had no experience in using smartphones for videoconferencing with people prior to this research; and 4) both residents and their family members agreed to participate in the study. A total of 32 participants in 5 NHs and 30 participants in 2 NHs were recruited for the intervention group and control group, respectively.

### Smartphone-based videoconferencing program

The smartphone-based videoconferencing program was designed to be used once a week, which was similar to the in-person visiting frequency for the majority of families [11, 12]. Yet, residents could videoconference with their family as many times as they wished. The goal of the duration of each videoconference was set for a minimum of 5 min [12, 14]. To achieve this goal, we provided some discussion topics to nurses and the participants, such as their meals, organised activities and ‘news’ on NH life. However, there was no enforcing or limitation on the duration. Videoconferencing with their family was recommended when participants were having meals or during activities to show their lives in the NH and to increase vivid interactions among the participants and their families. Trained research assistants or nursing staff recorded the number and duration of each videoconferencing session.

The mobile application, “LINE”, was the videoconferencing platform, in which the participant either used this technology independently or with the assistance of NH staff. This strategy was designed to achieve a real-time transmission between the participant and family. The intervention smartphone was a high-quality 8-in. HD (1280 × 800), chosen in recognition of residents’ physical limitations. The two sites (family and nursing home) were linked via Wideband Code Division Multiple Access (WCDMA) & Wi-Fi systems to achieve an appropriate video quality, providing a 3G connection (NT850/per month). A portable Android system with full 3G phone functionality allowed participants to be connected at any time of day. To obtain a better quality in the video image, high quality pictures were possible through a 2.0 Megapixels (MPs) front-facing camera and 5MP rear camera.

### Demographic variables

Demographic variables included participants’ age, gender, marital status, number of children, educational background, morbidity, duration of residency in the NH, and frequency of family visits. The number of family or friends who contacted residents and the frequency of these communications either by phone or in person during the previous week was also confirmed by asking nursing home staff to record the number of visits and phone calls made to the residents. Residents’ physical status was measured at baseline using the Barthel Index [20], which assesses performance of activities of daily living (ADLs). Outcome variables included subjective feelings of loneliness, depressive symptoms and QoL which were measured at baseline and repeated at 1, 3 and 6 months and compared to baseline scores.

### Outcome variables

The revised 10-item UCLA Loneliness Scale [21] measures subjective feelings of loneliness. Items are scored with a 4-point Likert scale from 0 (“I often feel this way”) to 3 (“I never feel this way”); total scores range from 20 to 80, with higher scores indicating a greater feeling of loneliness. This scale had a reliability of 0.87 in a study of older institutionalized adults [22]; in this study the Cronbach’s alpha was 0.91.

Depressive symptoms were measured with the 30-item Geriatric Depression Scale (GDS) [23]. Each item is scored with a yes/no response; possible scores range from 0 to 30, with higher scores indicating higher depressive symptoms. Previous studies in Taiwan have demonstrated the reliability and validity of the GDS have been supported in previous studies in Taiwan [12, 14]. In this study the Cronbach’s alpha was 0.91.

We measured QoL with the Taiwanese version of the short-form health survey, SF-36 [24]. The SF-36 is a self-report instrument that assesses eight dimensions of health-related QoL: physical functioning (PF), mobility restriction (MR), bodily pain (BP), general health (GH), vitality (V), social functioning (SF), emotional problems (EP) and mental health (MH). Each dimension is scored from 0 through to 100, with 100 representing good health and 0 representing poor health [25]. In a study of older adults with hip fracture the Cronbach’s alpha ranged from .59 to .96 [26]. In this study the Cronbach’s alpha ranged from 0.72 to 0.93.

### Procedure

Ethical consideration of the research was approved by the researchers’ Institutional Review Board. NHs in Taiwan do not have institutional review boards, therefore, we also obtained permission to conduct this study from the directors at each site prior to data collection. After this permission was granted, an announcement was posted at the NH entrance with details of our research purpose and procedure. We first asked NH staff to speak with residents who met our criteria and with their family members about their willingness to participate in the study. Residents and their families who were interested in taking part were contacted by research assistants who explained the goals and methods of the study. In addition, the potential risks, the entitlement of all participants to withdraw from the study at any time, the right to refuse to answer questions and the strategies used to protect anonymity and confidentiality were fully explained. After signing an informed consent document, we demonstrated how to use the smartphone and initiate videoconferencing to the residents and their family members in the interventional group. The residents and family members then made an appointment to use videoconferencing. Family members of residents in the intervention group could continue their in-person visits or telephone contacts as usual. Smartphones were available in the NHs for 6 months. Participants were able to use the smartphone in their own room or, at whichever location in the NH it was most convenient. The device could be used independently by the resident or with the assistance of the NH staff.

### Data analysis

All data were coded before being entered into a computer. Statistical analyses were performed using SPSS 24.0. Participants’ demographic data were analysed using descriptive statistics. Differences between the two groups were compared at four points (baseline, 1 month, 3 months and 6 months) using multiple linear regression of the generalized estimating equations (GEE) [27]. To examine the effect of the videoconferencing intervention on each variable, we constructed regression models which tested only one fixed effect.

## Results

### Participants

Sixty-two NH residents participated in this study. Demographic and clinical characteristics of the intervention and control group at baseline are shown in Table 1. The intervention group was significantly older than the control group (*p* < .001). The presence of hypertension and diabetes was significantly higher for the control group (*p* = .01 and *p* < .05, respectively).

**Table 1:** Baseline demographic and clinical characteristics of the intervention and control groups

Characteristic	Control group		Intervention group		t/χ^2^ (*p*)
	(*n* = 30)		(*n* = 32)		
Age, years (mean ± SD)	68.95	11.65	81.07	8.46	8.39 (< .001)^a^
Gender (n, %)					2.32 (0.13)^b^
Male	13	43.3	8	25	
Female	17	56.7	24	75	
Marital status (n, %)					9.38 (0.03)^b^
Single	9	30.0	2	6.3	
Married	4	13.3	12	37.4	
Divorced	4	13.3	2	6.3	
Widow/widower	13	43.4	16	50	
Number of children (mean ± SD)	2.10	2.07	3.61	1.86	3.04 (< .001)^a^
Education (n, %)					4.93 (0.43)^b^
None/illiterate	6	20.0	9	29.0	
Primary	10	33.3	13	41.9	
Junior High school	2	6.7	4	12.9	
Senior High school	9	30.0	3	9.7	
≥College	3	10.0	2	6.5	
Length of residency, days (mean ± SD)	391.33	813.83	292.6	828.40	−0.85 (0.39^a^
Weekly telephone calls					5.67 (0.02)^b^
No	29	96.7	24	75.0	
Yes	1	3.3	7	25.0	
Frequency of In-person visits (n, %)					9.63 (0.02)^b^
None/seldom	12	40	4	12.5	
Monthly	10	33.3	8	25.0	
Weekly (> 2times/month)	6	20	17	53.1	
Daily (> 5 times/week)	2	6.7	3	9.4	
Hypertension (n, %)					6.97 (0.01)^b^
No	7	23.3	18	56.2	
Yes	23	76.7	14	43.8	
Diabetes (n, %)					5.17 (0.02)^b^
No	12	40.0	22	68.8	
Yes	18	60.0	10	31.2	
Stroke (n, %)					0.52 (0.47)^b^
No	17	56.7	21	65.6	
Yes	13	43.3	11	34.4	

Note: *SD* standard deviation

^a^Independent samples t-test

^b^Chi-square test

The number of weekly phone calls and weekly in-person visits from family members was significantly greater (*p* < .05) in the intervention group than the control group.

During the 6-month study period, 12 participants in the intervention group withdrew from the study (3 declined to continue participating, 7 were discharged home, and 2 died), for an attrition rate of 37.5%. In the control group, 10 participants dropped out of the study (3 declined to continue participating, 4 were discharged home, and 3 died), for an attrition rate of 33.3%. There was no significant difference between participants who dropped out of the study and those who remained in age (t = 0.11, *p* = 0.74) or length of residency in the NH (t = 1.58, *p* = 0.21).

### Trajectory of videoconferencing intervention

During the 6 months of the study, the mean number of videoconferencing interactions in the first, third, and sixth months was 2.13 (SD = 2.52, range 0–12), 1.18 (SD = 1.34, range 0–15), and 1.62 (SD = 1.68, range 0–15), respectively. The mean number of videoconferencing interactions from the first to the sixth month did not differ significantly (F = 0.38, *p* = 0.68). Although the mean duration for videoconferencing interactions increased from the first month (270.48 s, range 0–1221 s) to the sixth month (367.31 s, range 0–1194 s), the increase was not statistically significant (F = 2.69, *p* = 0.07).

### Outcomes

Scores for outcome variables at baseline for both groups are shown in Table 2. The mean scores for feelings of loneliness were significantly different between groups (t = 0.87, *p* = 0.03). There was no significant difference between groups in mean scores for depressive symptoms (t = − 0.24, *p* = 0.81). Quality of life subscale scores were highest for emotional-role for both the control and intervention group (M = 84.44, SD = 29.99 and M = 81.25, SD = 32.72, respectively). The lowest scores were in physical functioning (M = 12.83, SD = 18.79 for the control group; M = 15.16, SD = 18.16 for the intervention group). The mean scores for physical-role (t = − 2.27, *p* = 0.03), pain (t = − 2.62, *p* = 0.01), and vitality (t = − 2.11, *p* = 0.04) were significantly different between groups.

**Table 2:** Quality of life, depressive symptoms, and feelings of loneliness for Control and Intervention groups at baseline^a^, 1-month^b^, 3-months^c^, and 6-months^d^ and differences between groups^1^

Variable	Control group		Intervention group		*t (p)*^*1*^
	Mean	SD	Mean	SD	
Feelings of loneliness					
Baseline	53.03	9.02	58.41	9.47	0.87 (0.03)
1-month	54.00	8.21	56.07	8.57	0.95 (0.34)
3-months	53.09	7.91	52.70	7.99	−0.17 (0.86)
6-months	52.50	6.98	53.08	8.62	0.21 (0.83)
Depressive symptoms					
Baseline	14.30	5.00	13.97	5.74	−0.24 (0.81)
1-month	14.40	4.97	13.80	4.71	−0.48 (0.63)
3-months	13.36	4.99	12.61	3.94	−0.57 (0.58)
6-months	13.65	5.40	13.62	3.23	−0.02 (0.98)
Quality of Life Subscales					
Physical function					
Baseline	12.83	18.79	15.16	18.16	0.49 (0.62)
1-month	13.33	19.22	14.83	17.44	0.32 (0.75)
3-months	13.64	12.17	16.52	18.98	0.60 (0.55)
6-months	15.25	12.62	10.77	11.15	−1.04 (0.31)
Physical role					
Baseline	62.50	37.57	41.41	35.70	−2.27 (0.03)
1-month	72.50	31.72	48.33	38.24	−2.66 (0.01)
3-months	76.14	30.35	68.48	35.53	−0.78 (0.44)
6-months	68.75	38.79	76.92	18.99	0.70 (0.49)
Emotional role					
Baseline	84.44	29.99	81.25	32.72	−0.41 (0.69)
1-month	91.11	21.32	87.78	26.96	−0.53 (0.60)
3-months	98.48	7.11	91.30	25.06	−1.29 (0.20)
6-months	93.33	13.68	89.74	21.01	−0.60 (0.56)
Social function					
Baseline	50.42	25.32	37.11	26.08	−2.00 (0.05)
1-month	49.58	26.16	39.17	25.16	−1.57 (0.12)
3-months	48.86	25.85	51.63	20.40	0.39 (0.69)
6-months	46.25	21.50	55.77	18.13	1.32 (0.20)
Pain					
Baseline	69.43	24.20	53.94	22.22	−2.62 (0.01)
1-month	68.77	24.75	57.63	21.74	−1.85 (0.07)
3-months	63.77	26.78	60.52	20.15	−0.46 (0.65)
6-months	61.65	24.39	59.92	19.09	− 0.22 (0.83)
Vitality					
Baseline	52.83	15.74	45.16	12.92	−2.11 (0.04)
1-month	52.50	15.24	46.33	11.74	−1.76 (0.08)
3-months	52.95	11.41	49.35	10.26	−1.12 (0.27)
6-months	46.75	13.01	50.38	7.49	0.91 (0.37)
Mental health					
Baseline	40.27	20.98	41.50	18.28	0.24 (0.81)
1-month	42.13	20.87	44.13	17.02	0.41 (0.69)
3-months	40.38	20.00	49.57	14.95	1.74 (0.09)
6-months	40.00	16.67	45.23	15.78	0.89 (0.38)
Perceived health					
Baseline	40.27	16.25	39.31	15.74	−0.25 (0.82)
1-month	37.83	17.17	39.37	14.12	0.38 (0.71)
3-months	34.14	15.23	39.30	15.44	1.13 (0.27)
6-months	33.45	12.02	34.38	13.38	0.21 (0.84)

Note: *SD* standard deviation; Quality of life subscales = mean subscale scores on the SF36; Depressive symptoms = mean scores on the Geriatric Depression Scale; Feelings of loneliness = mean scores on the UCLA Loneliness Scale

^1^Independent samples t-test

^a^Baseline: Control group *n* = 30, Intervention group *n* = 32

^b^1-month: Control group *n* = 30, Intervention group *n* = 30

^b^3-months: Control group *n* = 22, Intervention group *n* = 23

^d^6-months: Control group *n* = 20, Intervention group *n* = 20

The effect of the videoconferencing intervention on scores for feelings of loneliness and depressive symptoms is shown in Table 3. Mean scores for feelings of loneliness for the intervention group at 1-, 3- and 6-months decreased, on average, − 3.41 (95% CI − 1.78 to − 4.90), − 5.96 (95% CI − 3.14 to − 8.77), and − 7.50 (95% CI − 3.90 to − 11.10) units, respectively. Controlling for the effects of age and frequency of in-person family visits, these changes were significantly lower for the intervention group compared to the control group (all *p*-values < .001), demonstrating that feelings of loneliness decreased significantly at all three timepoints. However, there was no significant decrease from baseline in mean scores for depressive symptoms at any time point, and there were no significant differences between groups.

**Table 3:** Effect of videoconferencing intervention on feelings of loneliness, depressive symptoms, and quality of life at 1-, 3-, and 6-months (control group compared to baseline)

Variable	Unadjusted				Adjusted^b^			
	*β*	*SE*	*χ*^*2*^	*p*	*β*	*SE*	*χ*^*2*^	*p*
Feelings of loneliness^b^								
1-month x group	−3.41	0.79	18.48	< 0.001	−3.09	0.75	16.84	< .001
3-months x group	−5.96	1.44	17.24	< 0.001	−5.61	1.42	15.58	< .001
6-months x group	−7.50	1.84	16.70	< 0.001	−7.41	1.67	19.78	< .001
Depressive symptoms^b^								
1-month x group	−0.68	0.62	1.19	0.28	−0.47	0.63	0.57	0.45
3-months x group	−0.94	0.90	1.09	0.30	−0.73	0.90	0.65	0.42
6-months x group	−1.55	1.37	1.27	0.26	−1.52	1.28	1.41	0.24
Quality of Life Subscales^b^								
Physical function								
1-month x group	−0.21	0.79	0.07	0.79	−0.15	0.83	0.03	0.86
3-months x group	−0.77	1.48	0.27	0.60	−0.74	1.54	0.23	0.63
6-months x group	−1.89	1.73	1.20	0.27	−1.89	1.74	1.17	0.28
Physical-role								
1-month x group	−1.94	7.67	0.06	0.80	−2.06	7.81	0.07	0.79
3-months x group	13.30	9.20	2.09	0.15	12.91	9.32	1.92	0.17
6-months x group	36.49	13.09	7.78	0.01	35.47	13.08	7.36	0.007
Emotional-role								
1-month x group	1.00	7.53	0.02	0.89	0.72	7.71	0.01	0.93
3-months x group	−1.28	8.47	0.02	0.88	−1.65	8.60	0.04	0.85
6-months x group	2.21	8.35	0.07	0.79	1.63	8.46	0.04	0.85
Social function								
1-month x group	3.57	3.19	1.26	0.26	3.02	3.17	0.91	0.34
3-months x group	10.89	4.92	4.91	0.03	10.13	4.98	4.14	0.04
6-months x group	21.79	6.35	11.79	0.001	21.16	6.33	11.19	0.001
Pain								
1-month x group	4.62	4.05	1.30	0.26	4.39	4.10	1.15	0.28
3-months x group	10.83	6.19	3.06	0.08	10.49	6.24	2.83	0.09
6-months x group	16.71	6.14	7.40	0.01	16.42	6.10	7.24	0.007
Vitality								
1-month x group	2.30	2.38	0.94	0.33	2.05	2.35	0.76	0.39
3-months x group	5.20	2.72	3.66	0.06	4.91	2.69	3.33	0.07
6-months x group	13.11	3.72	12.41	< .001	12.97	3.67	12.48	< .001
Mental health								
1-month x group	1.54	2.10	0.53	0.47	0.86	2.13	0.16	0.69
3-months x group	8.03	2.78	8.34	0.00	7.46	2.74	7.40	0.007
6-months x group	7.07	4.33	2.66	0.10	7.12	4.08	3.05	0.08
Perceived health								
1-month x group	3.40	2.28	2.22	0.14	3.08	2.26	1.85	0.17
3-months x group	7.89	2.89	7.43	0.01	7.52	2.88	6.83	0.009
6-months x group	6.67	2.92	5.21	0.02	6.54	2.85	5.25	0.02

Note: Feelings of loneliness = mean scores on the UCLA Loneliness Scale, Depressive symptoms = mean scores on the Geriatric Depression Scale; Quality of life subscales = mean subscale scores on the SF36

^a^Adjusted for the effects of age and frequency of in-person visits

^b^Group: 0 = control group (reference group); 1 = intervention group

The effect of the videoconferencing intervention on quality of life subscale scores is shown in Table 3. Compared to the control group, mean scores for social function from baseline were significantly higher at 3-months (β = 10.89, *p* = .03, 95% CI 1.25 to 20.52) and 6-months (β = 21.79, *p* < .001, 95% CI 9.35 to 34.23); perceived health scores were also significantly higher at 3-months (β = 7.89, *p* = 0.01, 95% CI 2.22 to 13.56) and 6-months (β = 6.67, *p* = .02, 95% CI 0.94 to 12.41). Compared to controls, scores at 6-months for the intervention group were significantly higher than baseline for physical-role, vitality, and pain scores (β = 36.49, *p* = .01, 95% CI 10.84 to 62.14; β = 13.11, *p* < .001, 95% CI 5.81 to 20.40; and β = 16.71, *p* = .01, 95% CI 4.67 to 28.75, respectively). Mean subscale scores were also significantly higher than baseline for the intervention group for mental health at 3-months; scores increased, on average, 7.46 units (95% CI 2.49 to13.35) more than the control group (*p* = .004). Moreover, after controlling for age and frequency of in-person visits, scores for physical-role, vitality, and pain at 6-months, and social function and perceived health at 3- and 6-months differed significantly from baseline scores for the intervention group compared to the control group.

## Discussion

This study demonstrated that a smartphone-based videoconferencing intervention reduced feelings of loneliness in older NH residents at 1, 3 and 6 months. These results are similar to other studies showing that increasing the personal interactions of NH residents through the use of computer-based applications can significantly reduce feelings of loneliness [12, 14, 28].

There were some significant differences in baseline scores between the intervention and control group, which must be considered. The intervention group was significantly older than the control group. We have no data to explain this difference and can only speculate. One explanation for the age difference may be a result of the requirement that the NH residents’ adult children participate in the videoconferencing. The adult children willing to participate in videoconferencing may have had time because they were older, resulting in older NH residents in the intervention group. In addition to the age differences, baseline scores for feelings of loneliness were significantly higher in the intervention than control group. However, a recent study reported that although advanced age is associated with increased loneliness [29], age is only one of many contributing factors. A study reported older adults born in the 1920s had higher levels of loneliness than those born in the 1940s [30]. However, after controlling for age and frequency of in-person visits, the decrease in scores for feelings of loneliness from baseline to 6 months was significantly greater than the control group. Therefore, we believe the reduction in scores for feelings of loneliness is due to the videoconferencing intervention.

Our findings showed the videoconferencing intervention did not improve depressive symptoms of the NH residents at any timepoint. This result differs from previous reports, which demonstrated use of the Internet for communication with friends and family was associated with decreases in depression among NH residents [12, 14, 31]. These differences may be due differences in characteristics of the NH residents who participated. While two of the previous studies were conducted with NH residents [12, 14] the third study examined a large general population whose median age was 44 years [31]. Functional decline is an important predictor of depression [32], therefore, further studies are suggested to understand the effects of videoconferencing on different age cohorts. Characteristics of social networks, social support, negative interactions with a partner or close friend, and feelings of loneliness are reported to be predictors of depressive symptoms [33]. Therefore, there might be differences in social and clinical characteristics of our participants and those of the previous three studies.

Differences in the format of communicating via the Internet also might explain the differences in our findings from previous studies. Two studies [12, 14] conducted videoconferencing via computers or tablets and the third study [31] examined on-line communication. The screen of the smartphone in our study was smaller (8 in.) than desk-based computers (15.6 in.), our intervention was free to use, and the previous studies examined 3 months of weekly technology use. This is the first study to examine the effects of smartphone-based videoconferencing, therefore further study is suggested to understand the relationship of smartphone videoconferencing and depressive symptoms for older NH residents.

A novel feature of this study was to better understand the effects of videoconferencing on QoL. With the exception of a lower mean score for physical function, baseline subscale scores of the SF-36 in both the intervention and control groups is comparable to findings elsewhere [34, 35]. The lower scores for physical function compared to other studies might be explained by cultural differences in the reasons family members are placed in residential NHs Taiwanese society, which is often poor health [36].

The study found that videoconferencing positive effects on QoL indictors for pain, vitality and physiological health dimensions at 6-months, but not on physical function. Pain among older adults in NHs is common [37]. Participants may benefit from more support through videoconferencing and contact with their families because it is known that social interaction enhances pain management and is associated with pain reduction [37]. It may, thus, be important to include the use of videoconferencing technology as possible options during patient education and the development of care plans for pain management. However, information and communication technologies can indeed be valuable tools enabling better self-management and self-empowerment of pain [38].

Our research also revealed videoconferencing had positive effects on vitality at the 6-month timepoint. It has been suggested that allowing older NH residents to use communication and information technology improves their overall outlook; they report that they “feel young” or “become one of the modern generation” [39]. Due to the limitation of the sample size of this study, it is only possible to speculate whether technology of this kind influences vitality because participants feel more youthful. Future qualitative studies may be needed to better understand this relationship between using videoconferencing technology and the vitality of our participants.

The study revealed that the frequency of videoconferencing per month ranged from 1.18 to 2.13 times, which was lower than anticipated. The frequency, however, is comparable to previous computer-based videoconferencing research, which ranged from 1.22 to 1.46 times per month after the intervention period [14]. Such infrequent use indicates that as useful and convenient as videoconferencing may be, it cannot replace in-person visiting; and videoconferencing visiting can only be regarded as a “second-best option” to an in-person visit [34].

The participants in the intervention group had more children, which could explain the greater frequency of in-person visits and telephone calls compared to the control group. Thus, it is possible that family members of participants in the intervention group may have communicated by videoconferencing as an alternative to in-person visits and phone calls. Future videoconferencing interventions should assess frequency of weekly in-person visits to determine if there is a shift in the communication modality. We did not determine if adequate staff were available to help NH residents needing assistance with the smartphone-based videoconferencing equipment, which may have limited videoconferencing to participants who were more independent.

Our total attrition rate at the 6-month timepoint of 37.5% in the intervention group and 33.3% in the control group was higher than the 6-month attrition reported by two previous studies of older NH residents in Taiwan [14, 40]. Although the attrition rate in our study was high, an attrition rate of less than 40% is considered weak, based on the Effective Public Health Practice Project (EPHPP) quality assessment tool [41]. Therefore, we consider the attrition rate of this study acceptable. The higher attrition in the intervention group was primarily due to patients being discharged prior to completing the study, rather than dropping out; 7 out of 12 in the intervention group compared with 4 out of 10 in the control group. Whether videoconferencing contributes to discharge is something worthy of further study.

This study had some limitations. First, parent-child relationships and interactions in Asian cultures differ from Western cultures. This relational difference may limit the generalizability of the study results to Western countries. Second, we did not recruit cognitively impaired NH residents in our study. Whether smartphone videoconferencing can effectively increase QoL among older cognitively impaired residents has not been explored. Future studies should examine the use of this communication technology among older NH residents with cognitive impairments. Third, we did not distinguish between social and emotional loneliness. The UCLA Loneliness Scale measures subjective feelings of loneliness as a single global score. Future studies should be conducted which measure both social and emotional loneliness. Finally, our high attrition rate might limit the generalizability of our findings. Additional studies with a larger population is recommended.

The findings of our study demonstrate the effectiveness of a smartphone-based videoconferencing intervention on reducing feelings of loneliness and improving QoL for older NH residents. We suggest future research be conducted to better understand how Internet technology can improve the emotional health and QoL for older NH residents. Studies should include examining the effect of videoconferencing using different combinations of media-based technologies and program designs; variables should include not only loneliness and depressive symptoms, but also stratification of age, number of children and/or family members, and cognitive status. Qualitative studies could add additional information regarding the response of NH residents to such interventions. Finally, cultural differences should be explored to determine if interventions should be tailored to match the cultural setting of the NH residents. Such explorative studies could provide important considerations for the development of interventions to enhance health of the older population within the NH setting.

## Conclusions

This study demonstrated that a smartphone-based videoconferencing intervention reduced feelings of loneliness at 1-, 3-, and 6-months and improved variables of QoL. The findings suggest smartphone-based videoconferencing is a feasible option for enhancing the care of older NH residents by improving communication with their family members. Nursing home managers and care staff could incorporate videoconferencing technology as a standard part of NH resident care. Videoconferencing could be an important intervention for patients who are at risk for loneliness or have symptoms associated with a poor QoL. Such changes in the care of NH residents may be especially important for healthcare professionals working with populations of East Asian nationality or descent.

## Data Availability

The data analyzed during the current study are available from the corresponding author on reasonable request.

## Abbreviations

ADLs: activities of daily living
BP: bodily pain
EP: emotional problems
GDS: Geriatric Depression Scale
GEE: generalized estimating eqs.
GH: general health
MH: mental health
MMSE: Mini-Mental State Examination
MR: mobility restriction
NH: nursing home
PF: physical functioning
QoL: quality of life
SF: social functioning
V: vitality
